# Extent of sediment concentration trends associated with climate and human factors across global rivers

**DOI:** 10.1038/s41598-026-47267-2

**Published:** 2026-04-03

**Authors:** Rajaram Prajapati, John Gardner, Punwath Prum

**Affiliations:** 1 Department of Geology and Environmental Science, University of Pittsburgh, Pittsburgh, PA USA; 2 SmartPhones4Water (S4W), Chico, CA USA; 3 Department of Earth, Marine and Environmental Sciences, University of North Carolina at Chapel Hill, Chapel Hill, NC USA

**Keywords:** Suspended sediment, Satellite remote sensing, Rivers, Landsat, Global change, Climate sciences, Ecology, Ecology, Environmental sciences, Hydrology

## Abstract

Suspended sediment concentration (SSC) is a key indicator of river ecosystems, influencing aquatic habitat quality, biogeochemical cycling, reservoir sustainability, and the persistence of downstream coastal land. Despite its global significance, long-term assessments of SSC trends have been limited in spatial scope. Leveraging > 88,000,000 satellite-derived SSC estimates, we analyzed trends over a 38-year period (1984–2022) across > 200,000 river segments globally. Our analysis reveals significant SSC trends in one-third of rivers, with 27% exhibiting declines and 7% showing increases. Basins with more widespread declining SSC trends are in temperate and arid regions and the extent of change is primarily associated with dam regulation and forest recovery. In contrast, increasing trends are concentrated in tropical basins, where deforestation and high rainfall causes erosion. These findings highlight the value of a spatially and temporally coherent, global riverine SSC database for documenting and pinpointing environmental change in unmonitored rivers.

**Supplementary Information:**

The online version contains supplementary material available at 10.1038/s41598-026-47267-2.

## Introduction

The global sediment cycle is being profoundly altered by human activities and this is recorded in rivers^1,2^. Land use change has caused more landscape erosion over the last 100 years than over the last 0.5 billion years^3^, but dams trap ~ 25–50% of the global riverine sediment that would be transported to the ocean^4^ resulting in complex changes in sediment concentration and transport within river corridors. We focus on suspended sediment as a majority of the total riverine sediment flux moves in suspension in large rivers^5^. Suspended sediment concentration (SSC) and transport play key roles in global biogeochemical cycles and maintaining healthy riverine and coastal ecosystems^6,7^. However, long records of suspended sediment concentration are rare^8^. We need globally consistent, open-source, and long-term records of riverine suspended sediment to better understand how the sediment cycle is responding to local and global change.

Recent advances in satellite remote sensing and computational approaches have revolutionized our ability to monitor SSC over multiple decades^9^ and draw insights at basin to global scales. The longest running satellite mission, Landsat, has been used to quantify trends in sediment flux at the outlet of 400 river basins^10^, quantify the stability in shape of downstream SSC profiles^11^, and link cryosphere changes to SSC response across the Tibetan plateau^12^. Recent studies quantifying global changes in SSC, sediment flux, and their drivers have found contrasting results. Work focusing on the outlet of 414 major rivers suggested declines in SSC and flux are more common than increasing trends and attributed declines to dams and increasing trends to deforestation^13^. Using a similar approach, Sun et al. found there is a net global increase in sediment flux from a similar set of ~ 400 major river outlets and they extensively mapped SSC in upstream rivers finding 43% of river segments showed increasing SSC while 25% showed decreasing SSC^14^. Increasing trends were attributed to changes in precipitation expressed as rainfall erosivity, an integrated measure of the potential of rainfall to cause soil erosion^15,16^. However, the extent of SSC changes across river basins remains poorly understood, and the combined influence of human, climate, and landscape factors on these changes has not been systematically evaluated. Further, open-source SSC databases for all Landsat visible rivers over the entire Landsat 5, 7, 8, record remain limited.

Here, we present a global database of riverine SSC, called GloRivSed, that integrates Landsat-derived SSC data with river hydrography providing a spatially explicit and topologically connected perspective on SSC. We quantified 38-year trends (1984–2022) in SSC across Earth’s rivers > 50 m wide. We focused on the extent of SSC changes as there is considerable past work dedicated to sediment flux at basin outlets^13,17^. Focusing on 25 major river basins that are well represented within the Surface Water and Ocean Topography River Database (SWORD)^18^, we examined human, climate, and landscape factors associated with differences in the basin-wide extent of SSC trends. Specifically, we related changes in land use, changes in dams, and basin characteristics with the extent of SSC trends. We asked (1) How widespread are SSC trends across global rivers? (2) Why do some basins have more widespread SSC changes than others?

## Results and discussion

### Global riverine SSC database (GloRivSed)

**Fig. 1:**
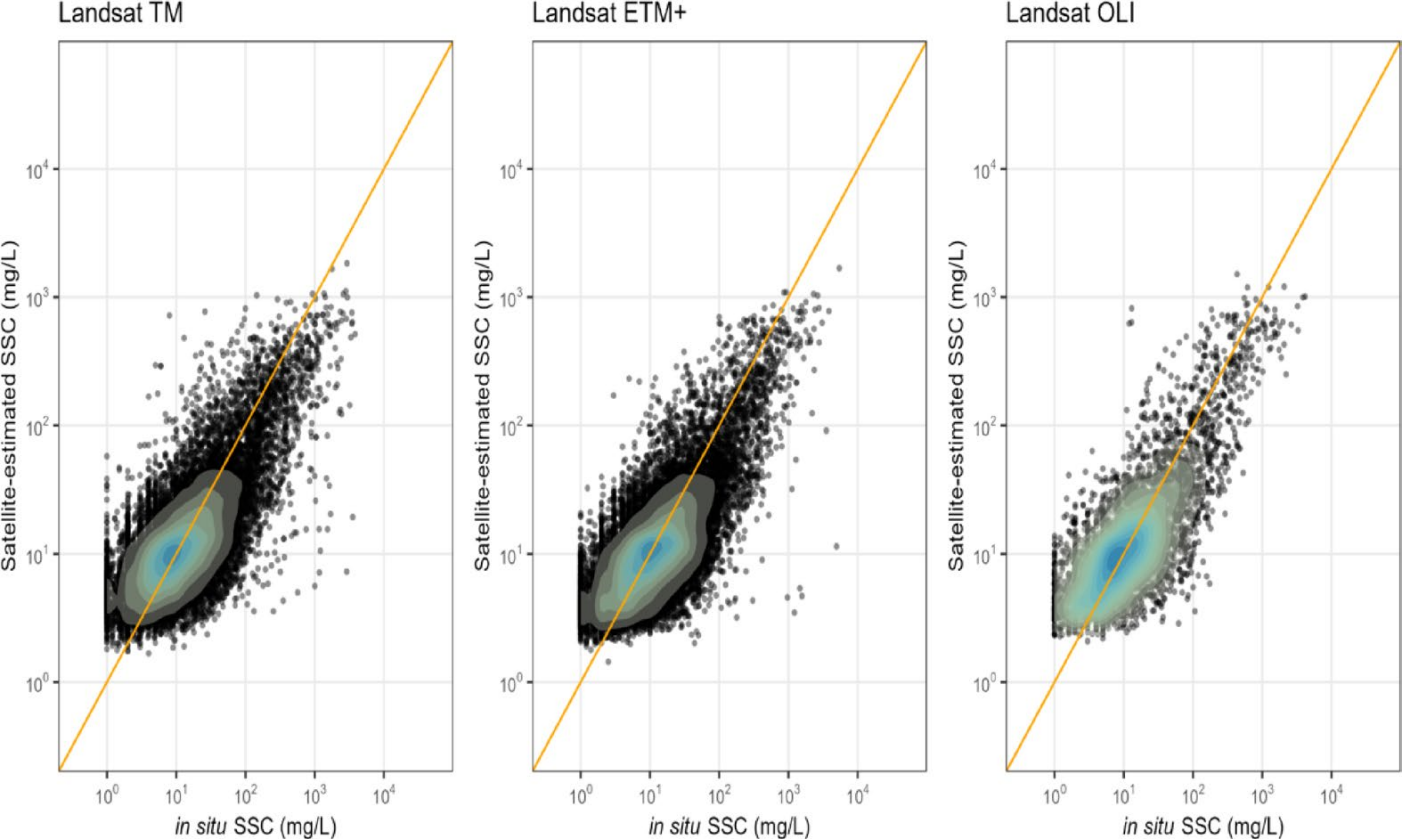
In situ versus satellite-estimated suspended sediment concentration (SSC) for three Landsat sensors. Each subplot displays SSC values on a log-log scale with density contours overlaid on individual observations. The orange dashed line represents the 1:1 reference line.

**Fig. 2:**
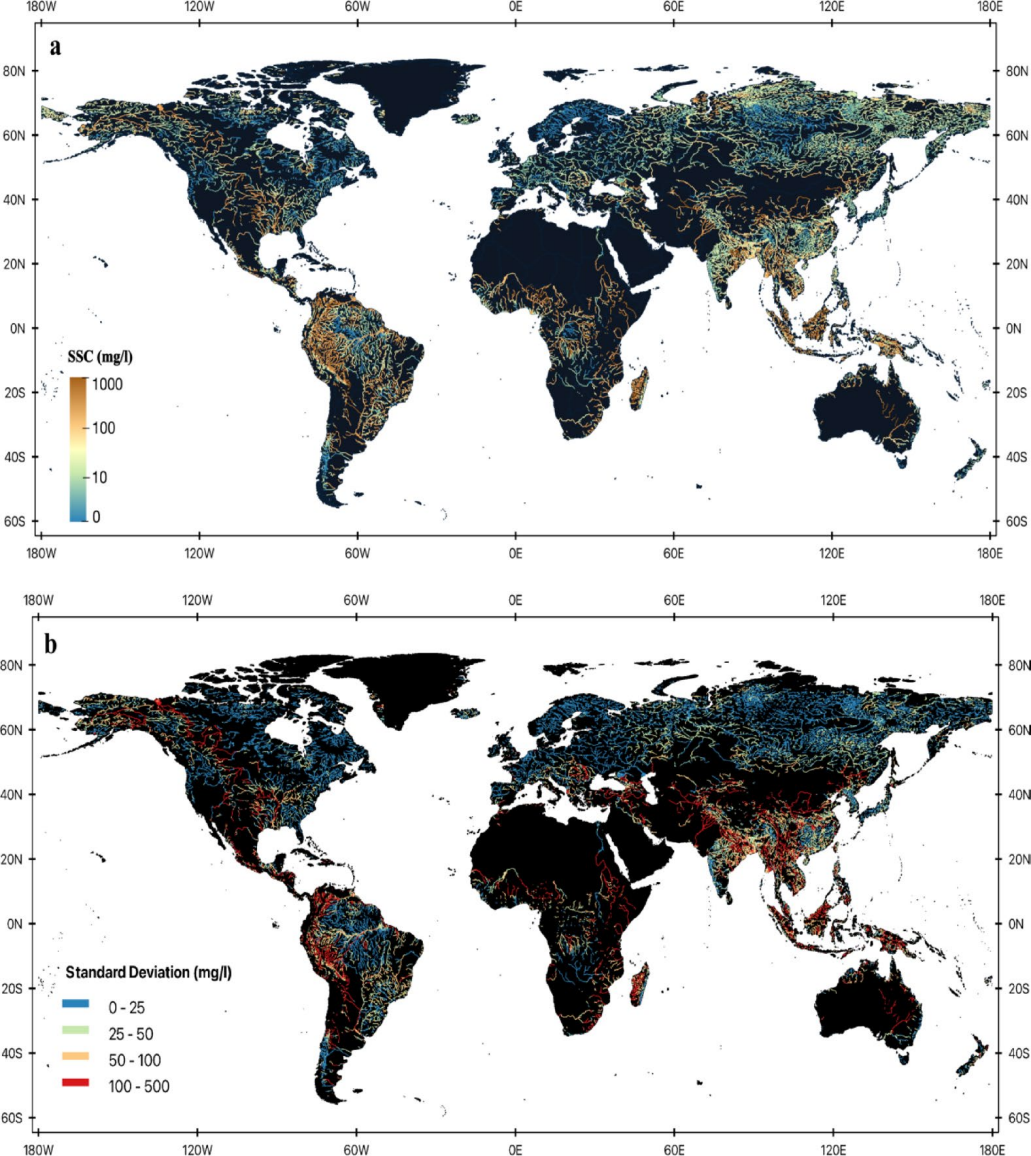
Global distribution of long-term mean suspended sediment concentration (SSC) at the river reach level, derived from Landsat imagery spanning 1984 to 2022. (**a**) Mean SSC values across 125,551 SWORD river reaches, presented on a base-10 logarithmic scale to capture the wide range of concentrations. Note, some regions have data gaps in Landsat observations due to lack of global coverage during Landsat 5^25^ as well as seasonal cloud cover. (**b**) Standard deviation of SSC over the same period, illustrating temporal variability at each reach. The map was generated by the authors using QGIS 3.34 (https://www.qgis.org) and does not require any permissions.

Our goal was to produce a coherent riverine SSC database that is interpretable over space along a river and over multiple decades. To achieve this goal, we highlight key advances and differences with previous work. (1) A single generalizable SSC algorithm built using spatial-temporal cross validation: Existing global approaches use 2–7 different algorithms for different rivers typically based on optical water type, or water color, and/or the model was not thoroughly tested on hold-out data and validated over space^19,20^. While multiple algorithms may improve error metrics, using multiple algorithms can produce abrupt spatial discontinuities in SSC when predicting over space^21^. Therefore, we applied a single algorithm that includes features representing water color and trained on data from rivers representing a wide range of water color conditions. Further, we used spatial-temporal cross validation to ensure model transferability and reduce overfitting^22^. The globally validated and tested SSC model is presented in a companion work, Prum et al.^23^ and summarized in the methods section here. (2) Inclusion of Landsat 5, 7, 8, and extensible to Landsat 9, using a sensor harmonization specific to water: To our knowledge, no studies have produced riverine SSC data across Landsat 5–8 nor harmonized surface reflectance (Rs) for more accurate time series analysis. (3) Sharing full code stacks and full global SSC database over the same footprint as SWORD reaches: Sun et al.^14^ also produced a reach level SSC linked to GRWL^24^, a SWORD predecessor, including monthly SSC values limited to rivers wider than 120 m. Our database includes all individual SSC observations in rivers down to 50 m wide, and it is joined to SWOT SWORD of Science products, and formatted for ease of use by the scientific community (Figs. 1 and 2).

### Global trends in riverine SSC

**Fig. 3:**
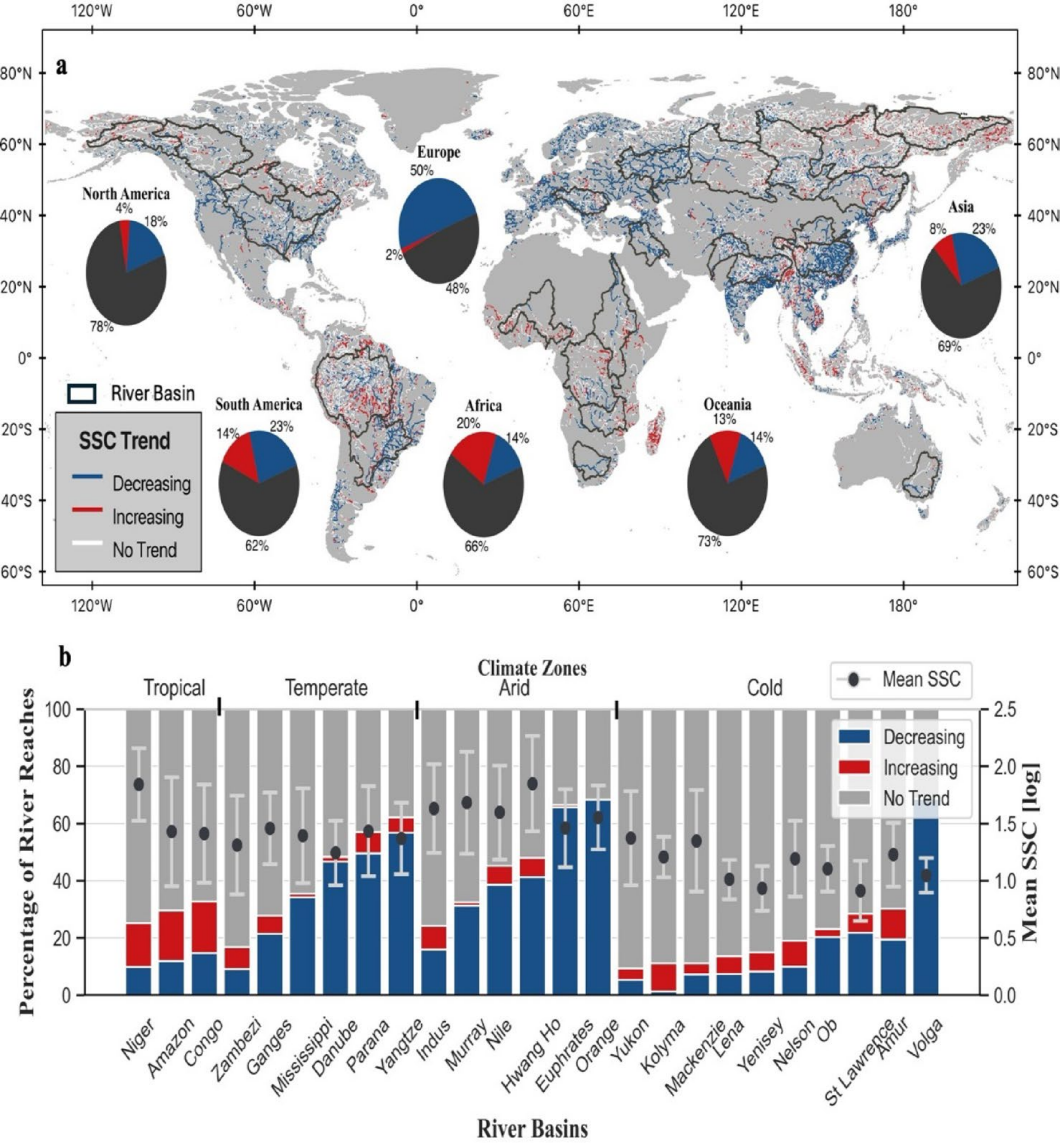
Long-term trends across global rivers. (**A**) Annual mean SSC trend identified using the Mann-Kendall test (p-value < 0.05 for significance). River reaches with increasing SSC trends (red lines), river reaches with decreasing SSC trends (blue lines), river reaches with no statistically significant trends (white lines) and major river basins outlined (black line). Major basins were used to identify factors over the extent of change. Pie charts represent the percentage of river reaches with increasing (red), decreasing (blue) and no statistically significant trends (black) in each continent. (**B**) Boxplot showing percentage of river reaches with increasing (red), decreasing(blue) and no statistically significant trends (black) in major river basins classified by their dominant climate type. Secondary y-axis shows the basin average SSC (in log10 space) and standard deviation as the error bars. The map was generated by the authors using QGIS 3.34 (https://www.qgis.org) and does not require any permissions.

Over the past 38 years, one-third of large rivers show significant decreasing or increasing SSC trends (*p* < 0.05). Notably, 80% of rivers with significant temporal trends (i.e. 470,000 km) show a decrease in SSC (27% of all rivers), while 20% (i.e. 127,000 km) have experienced increases (7% of all rivers) (Fig. 3). Europe is particularly noteworthy, with over half of its rivers (52%) showing significant temporal trends, and a remarkable 96% (73,000 km) of these trends showing declining SSC. Similarly, in Asia and North America, more than 80% of rivers (Asia: 184,000 km, North America: 70,000 km) with significant temporal trends show decreases in SSC. In contrast, South America and Oceania display a more complex picture. Although two-thirds of rivers (South America: 73,000 km, Oceania: 14,000 km) with significant trends show decreases, a notable proportion also exhibit increasing SSC (South America: 34,000 km [11%], Oceania: 7,000 km [9%]). Africa presents a unique case, with around 40% of its rivers exhibiting significant temporal trends, half of these with decreasing SSC (24,000 km).

**Fig. 4:**
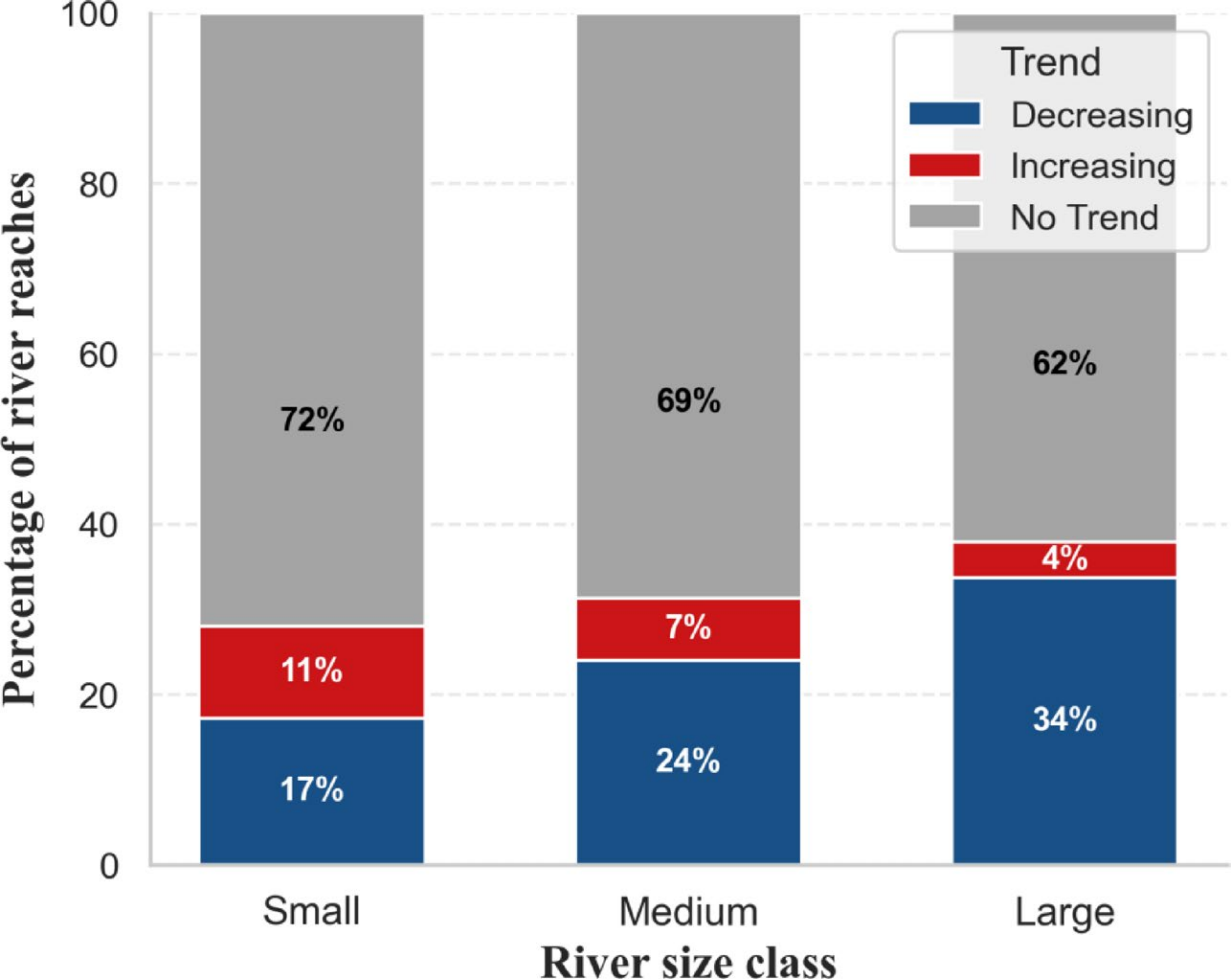
Distribution of SSC trends across river size classes. Small rivers show a higher proportion of increasing trends, while large rivers are dominated by decreasing trends; most reaches across all size classes exhibit no significant trend.

To further examine whether SSC trends vary across river hierarchy, we analyzed SSC trends according to river width classes, which serve as a proxy for river size and network position. River width is correlated with drainage area and stream order in river networks and therefore provides a practical proxy for river hierarchy at the global scale^26,27^. River reaches were classified into three width classes (50–100 m, 100–300 m, and > 300 m), representing relatively small, medium, and large rivers within the dataset. We then evaluated the proportion of increasing and decreasing SSC trends across these classes (Fig. 4). The results show systematic differences in SSC trend direction across river sizes: large rivers exhibit the highest proportion of decreasing SSC trends, whereas increasing trends occur relatively more frequently in smaller rivers. Specifically, decreasing SSC trends occur in 34% of large rivers, compared with 24% of medium rivers and 17% of small rivers, while increasing trends are observed in 11% of small rivers, 7% of medium rivers, and 4% of large rivers. This pattern is consistent with the expected influence of sediment trapping by reservoirs in major river systems, while smaller rivers may respond more strongly to climate-driven erosion and land-surface disturbances. Basin-level comparisons further indicate that this pattern is broadly consistent across basins rather than being driven by a small number of large river systems.

### Explaining the extent of SSC trends across major river basins

We tested a wide range of basin-scale climate, human, and landscape factors across 25 major basins and 214 sub-basins for correlations with the fraction of the river basin with decreasing or increasing trends. Several factors were temporal, including percent change in land cover (2000–2022) and change in Degree of Regulation (DOR), which captures dam expansion over the broader SSC record period (1984–2022). Among the major river basins, the extent of decreasing SSC trends ranged from 1.3% to 68.7% and was significantly associated with trends in rainfall, percent change in forest and shrubland cover, and temporal Degree of Regulation (DOR) (Table 1). Using multiple linear regression (MLR), these four variables together explained 45.2% of the variance (adjusted R² = 0.452) in the fraction of rivers declining in SSC with change in DOR and change in forest cover having the strongest effect. Specifically, for every unit increase in DOR over time, holding all other independent variables constant, the fraction of rivers with decreasing SSC trends increased by 9.3% points (*p* = 0.005), and a 1% increase in forest cover over time resulted in a 7.3% points increase in the fraction of rivers with decreasing SSC trends (*p* = 0.022). Statistically significant positive correlations (basin *r* = 0.60, sub basin *r* = 0.58) between the change in DOR and the fraction of river reaches with decreasing SSC trends were observed at both basin and sub-basin levels, suggesting that increased dam regulation is associated with SSC decline across spatial scales. The extent of increasing SSC trends could not be robustly explained using MLR likely due to smaller sample size of increasing trends and non-linearities and lags in fluvial response to climatic and land use change that could increase SSC^28^. However, correlation analysis highlighted rainfall erosivity (basin *r* = 0.62), average rainfall (basin *r* = 0.52), and decreases in forest cover (basin *r* = −0.39) as key associations with the fraction of rivers with increasing SSC trends (Table 1). Lithologic and topographic factors were not significantly correlated with the extent of decreasing or increasing SSC changes (Table 1), suggesting human and climatic factors are more important for explaining the extent of change regardless of the underlying geology.

Sub-basin scale analysis agreed with basin scale analysis of factors related to the fraction of increasing or decreasing SSC trends. Although sample size was larger at the sub-basin scale (*n* = 214), the greater variability among sub-basins reduced model fit and limited the effectiveness of multivariate regression, therefore, we emphasize correlation analyses. Sub-basin correlation analysis (*n* = 214) confirmed that the same associations identified at the basin scale remain significant at finer spatial resolution. The fraction of reaches with decreasing SSC trends was positively correlated with both forest cover change (*r* = 0.46, R² = 0.21, *p* < 0.001) and change in DOR (*r* = 0.48, R² = 0.23, *p* < 0.001), while rainfall erosivity was strongly associated with the fraction of increasing SSC trends (*r* = 0.59, R² = 0.34, *p* < 0.001). Stratifying the river basins by their dominant climate-zone suggests basin scale response may differ across climate zones, but the reduced sample size and statistical power of climate-zone specific analysis should not be interpreted quantitatively. These findings reinforce that change in dam regulation, forest cover change, and rainfall erosivity are dominant and spatially persistent factors that may explain why some basins have more SSC trends than others, even when tested at a finer, sub-basin scale.

**Table 1:** Summary of Multiple Linear Regression (MLR) results assessing the relationship between selected variables and the percentage of river reaches with decreasing SSC trend. The table presents the estimated coefficients, standard errors, t-statistics, and p-values for each variable included in the model.

Variables	Coefficient	Standard error	t-statistic	*p*-value
Constant	27.3731	3.297	8.303	0.000
Forest cover change (%)	8.2983	3.333	2.4490	0.022
DOR change (%)	9.8129	3.344	2.934	0.008
Rainfall trend (mm/year)	−5.4023	3.358	−1.609	0.123
Shrubland change (%)	1.7325	3.374	0.514	0.613

Adjusted R-squared: 0.452, F-statistic: 5.947 (p-value = 0.00254).

**Fig. 5:**
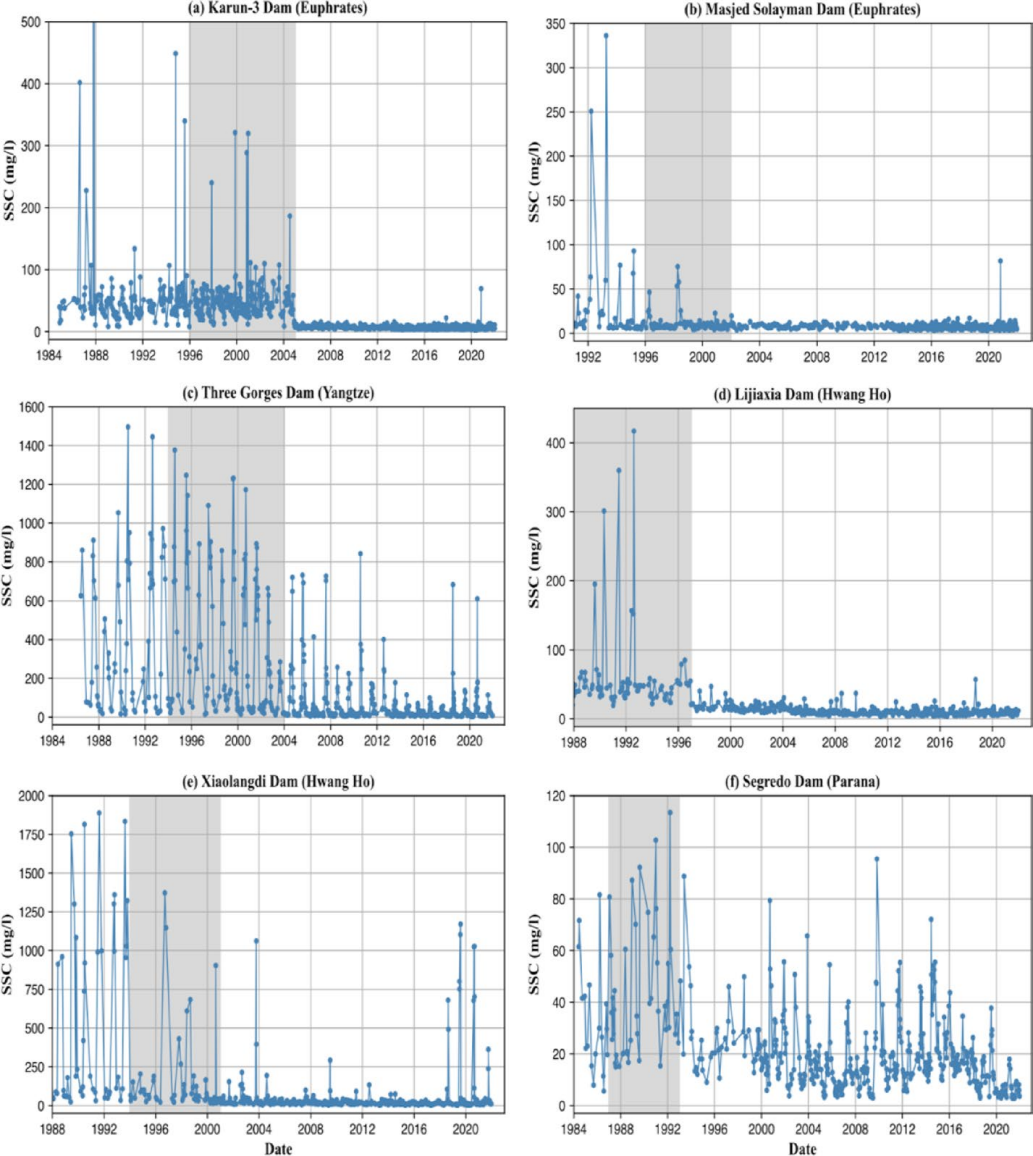
SSC time series downstream of select dams located in temperate and arid climate zones. The gray shaded period denotes the dam construction period. A noticeable reduction in SSC is observed after dam completion, indicating substantial sediment trapping and disruption of natural sediment flow downstream.

The dominant climate zone is useful for interpreting the relationship between growing dam storage and the extent of SSC changes. Temperate and arid climate zones contain the highest proportion of significant SSC trends (~ 50%) which are dominated by declines accounting for 90% of the significant trends (Fig. 3b). This is related to severe dam regulation (DOR > 75%). In temperate regions (mean DOR = 73%), dams are crucial for regulating seasonal water fluctuations, mitigating flood risks, and supporting growing water demands^29^. Rivers in arid regions are highly regulated (mean DOR = 496%) to maintain water supply during dry periods^30^. High DOR and efficient sediment trapping of individual dams in arid regions, due to large reservoir capacities and lower river discharge^4,31^, work together to decrease SSC. For instance, the Euphrates and Orange River basins (arid zones) show nearly 100% of significant trends are declining trends. Our findings are consistent with previous studies reporting SSC declines in dam-impacted regions of Asia and southern Africa^14,32^. While Dethier et al^13^. suggested the impact of post-1984 dams may be diminishing due to pre-existing regulation, our basin-level results indicate that in many arid and temperate rivers recent dam expansion continues to drive significant SSC reductions (Figure S4 and Fig. 5). During this period, around 90% of the 376 new dams built in the select river basins were located in temperate (66%) and arid (21%) zones^33^, leading to a 30% rise in overall storage capacity.

**Fig. 6:**
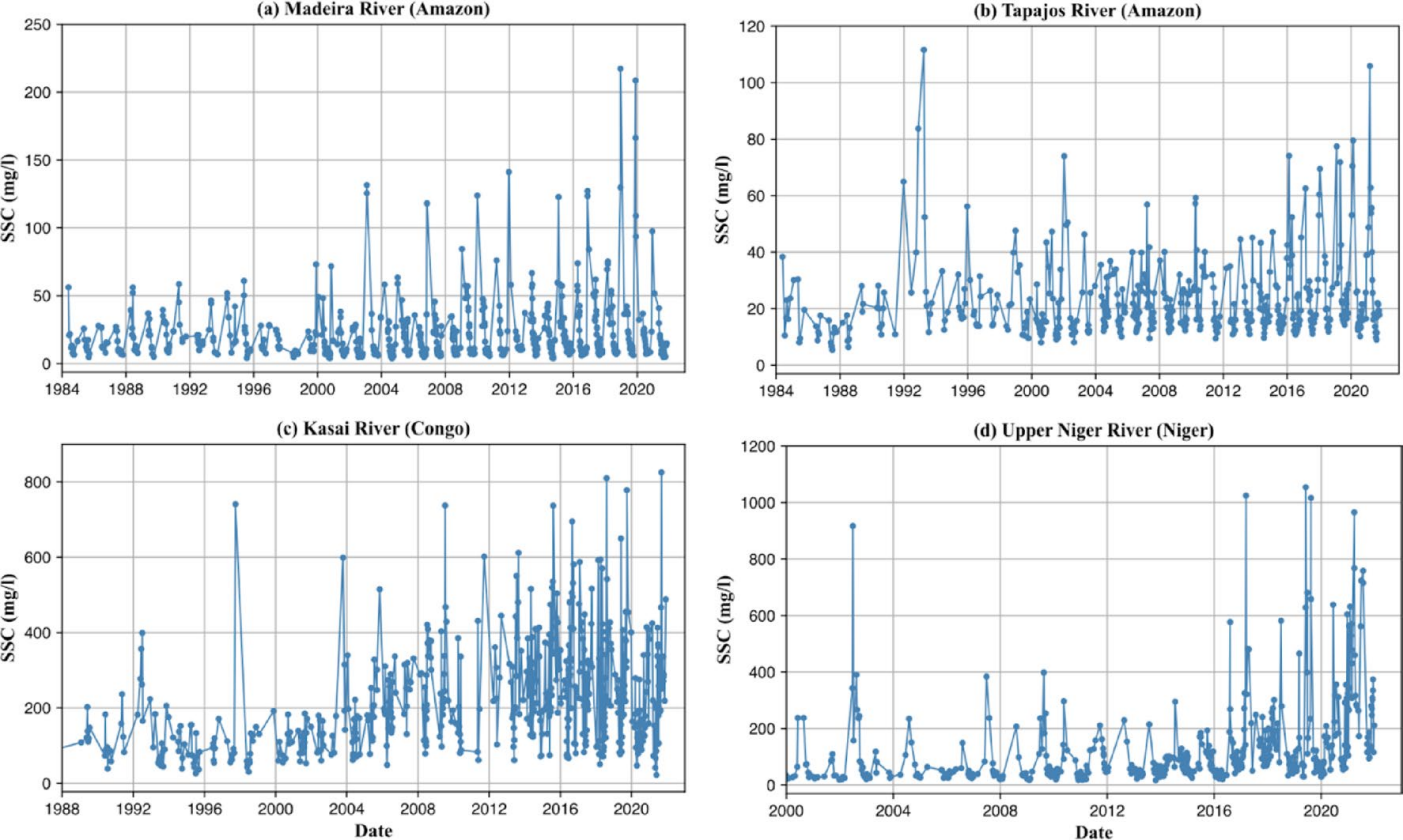
SSC time series in select rivers of tropical climate zone. A noticeable increase in SSC is observed over time, which may be attributed to deforestation, accelerated land cover change and mining activities.

Changes in forest cover influence the extent of SSC trends in both directions. We found a statistically significant positive correlation (basin *r* = 0.61, sub basin *r* = 0.60) between forest cover gain and the extent of decreasing SSC trends in both basin and sub-basin levels, and a negative correlation (basin *r* = − 0.51) between forest cover loss and increasing SSC trends at the basin level (when excluding 8 basins with minimal, < 5% forest cover). The impact of deforestation on SSC is tied to its magnitude, where increases in SSC are positively correlated with the rate of deforestation^34^. For example, in the Yangtze basin, a 6.5% increase in forest cover may contribute to widespread SSC decreases in 94% of river reaches with significant temporal trends (56% of total river reaches). Conversely, in the Amazon basin, a 4% forest cover decrease may contribute to SSC increases in over 40% of river reaches with significant trends (12% of total river reaches). These results are consistent with previous findings linking SSC increases in tropical rivers to direct human activities such as deforestation and mining (Fig. 6)^13^, and associating low forest cover basins with higher sediment yields^14,35,36^.

Rainfall erosivity was the most significant factor associated with the extent of increasing SSC trends. We focused on long-term mean erosivity to better represent the baseline erosive force that drives sediment production over the study period. Fluvial response to climate trends over the last few decades is notoriously difficult to quantify and isolate due to gradual climate change, lagged fluvial sediment response^34^, and compounding factors such human modification of rivers and their landscapes^13,28,37^. The correlation analysis reveals a strong association (basin *r* = 0.75, sub basin *r* = 0.59) between rainfall erosivity and the fraction of river reaches with increasing SSC trends in both basin and sub-basin levels, excluding river basins from cold climate zones where snowmelt and freeze-thaw cycles, rather than rainfall, dominate erosion processes^12^. Our analysis suggests that river basins in tropical regions with high mean rainfall erosivity (> 8000 MJ mm ha⁻¹ h⁻¹ yr⁻¹) and limited dam regulation may experience significant soil detachment and transport, leading to elevated SSC over time. This pattern is consistent with previous work showing rainfall erosivity is a key driver of the rate of SSC increase in basins with minimal dam influence^14^.

### Limitations and data use recommendations

While our analysis of SSC trends provides valuable insights into basin-scale sediment dynamics, several limitations must be acknowledged. First, data gaps and uneven temporal distribution of SSC observations across different river reaches could introduce biases, particularly in regions with limited monitoring coverage due to seasonal cloud cover. To minimize these effects, we applied minimum thresholds for the number of observations and record length required for a representative time series for trend analysis (see Methods: Data Analysis), thereby reducing the influence of sparsely sampled reaches. The non-parametric Mann-Kendall test, while robust against outliers, cannot detect subtle or non-monotonic trends, therefore our analysis represents river reaches with consistent long-term trends, not breaks or changes in the trends over time. For example, disturbances such as deforestation may produce short-term increases in sediment concentrations that later stabilize or decline as landscapes adjust, which would not necessarily be captured by a monotonic trend framework. These limitations can be addressed in future work that leverages the full time series in GloRivSed with observations up to every 8–16 days to understand local context of individual basins and rivers and variability over shorter time scales.

GloRivSed is best suited for applications that emphasize relative comparisons of SSC, rather than precise absolute values. It is particularly well-suited for analyzing long-term trends in SSC at the reach or basin scale, identifying before–after changes related to significant intervention such as dam construction, deforestation etc., and exploring spatial variability along a river continuum, across regions, climate zones, or geomorphic settings. The database is also valuable for mapping SSC hotspots, conducting global or continental-scale synthesis, and supporting basin-scale planning where a consistent SSC data source across a large area is essential. GloRivSed can be integrated with complementary global datasets such as discharge, rainfall, land use, and rainfall erosivity to investigate the drivers of sediment dynamics and to support large-scale exploratory modeling of sediment transport patterns. However, users should exercise caution when applying the dataset in contexts that require highly accurate absolute SSC values in specific locations—such as sediment load estimations or compliance with water quality thresholds—without local calibration or corrections for depth-integrated SSC. Similarly, it is not intended for event-scale analyses requiring high temporal resolution. Used appropriately, GloRivSed can serve as a powerful resource for advancing our understanding of how riverine sediment dynamics over time and space.

## Conclusions

We present a first analysis GloRivSed, a comprehensive global riverine SSC database totaling 88,436,343 observations over 224,718 river reaches from 1984 to 2022. We found declining trends are more prevalent than increasing trends across Earth’s large rivers. The basin-wide extent of SSC trends is highly sensitive to changes in forest cover and changes in river regulation (DOR) with small changes in DOR and forest cover associated with significant growth or contraction in the extent of declining SSC trends within a basin. Sub-basin analyses reinforce these patterns, showing that rainfall erosivity, forest recovery, and dam regulation are associated with the extent of SSC declines at finer spatial scales. The sensitivity of extent of SSC change highlights the utility of spatial data, like GloRivSed, as basin wide trend maps can detect localized change. GloRivSed is a publicly accessible, coherent database for assessing SSC changes over space and time enabling analysis of sediment dynamics across river networks and supporting applications in river geomorphology, aquatic ecology, and sediment management.

## Materials and methods

### Suspended sediment concentration database

To build GloRivSed, we generated a Landsat surface reflectance (Rs) database over the same footprint as a vector database of global river reaches using the SWOT River Database (SWORD)^18^ and applied our global SSC algorithm^23^. We used the USGS Collection 1 Tier 1 Landsat Surface Reflectance product (T1-SR) because it was the most up-to-date product available at the time of analysis, prior to the recent release of Collection 2^38–40^. We will release a Landsat Collection 2 update for GloRivSed. The same image processing workflow was applied to both the SWORD Rs database and match-up database, or coincident satellite and field measurements, used to train and test our SSC model with one key difference being the SWORD Rs database is based on river reaches (mean length = 8 km) rather than points used for generating match-ups.

**Fig. 7:**
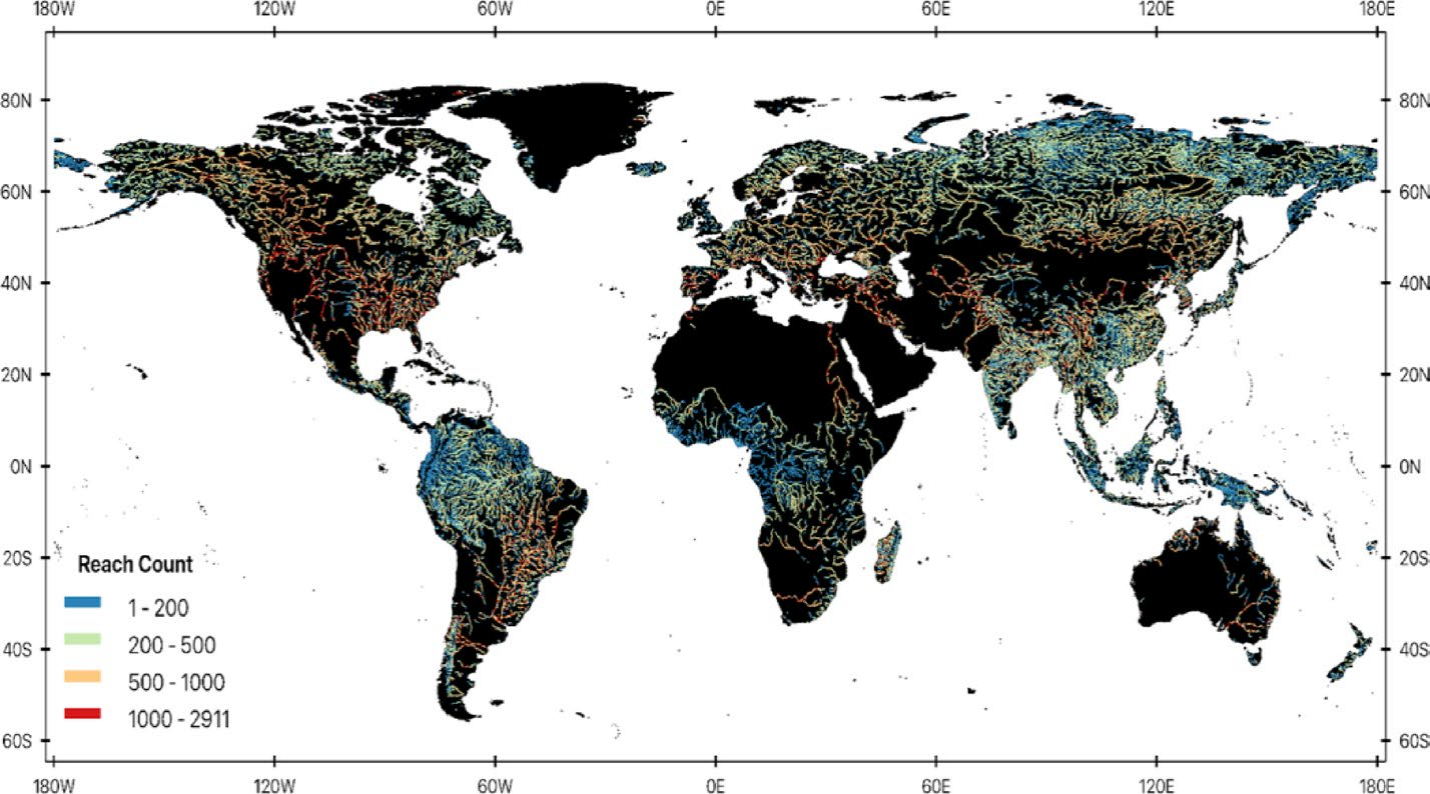
Global distribution of SWORD river reaches showing the number of Landsat-derived surface reflectance observations used to estimate SSC for each reach (reach count) during 1984–2022. The map was generated by the authors using QGIS 3.34 (https://www.qgis.org) and does not require any permissions.

Image analysis was performed in Google Earth Engine via its python API based on published methods designed specifically for rivers. Our approach for extracting Rs accounts for fluctuating river position, size, topographic shadows, clouds, cloud shadow, snow, ice, and river obstructions to focus on high quality, open water river channel pixels^41^. Over 88,436,343 Rs records were extracted over 224,718 SWORD river reaches from 1984 to 2022 across Landsat 5, 7 and 8 (Fig. 7). The dynamic surface water extent (DSWE) algorithm^42^ was used to identify high probability open water river pixels and mask vegetated water in each image. The Landsat quality assessment band generated by FMask was used to mask cloud, cloud shadow, snow, and ice^43,44^. To account for differences in band centers and ranges of different Landsat sensors to enable time series analysis, we harmonized Rs from Landsat 5 and 8 to match Landsat 7 following Gardner et al^41^. and Topp et al^45^..

Here, we summarise the SSC model presented in Prum et al.^23^,. A match-up database of coincident satellite Rs and field SSC observations was made by aggregating public data sources from around the world. Field SSC data sources include the Water Quality Portal (National Water Quality Monitoring Council)^46^, Waterbase (European Environment Agency)^47^, South America by Hydro-geochemistry of the Amazonian Basin or Hybam^48^, Brazilian Water Agency (Hidroweb)^49^, Global Environment Monitoring System for Freshwater database (GEMStat)^19,50^, and Global River Chemistry database (Glorich)^51^. Using these matchups, our SSC model, to our knowledge, leverages the largest number of training and testing data to date with 190,558 for training and 49,666 for testing (Fig. 7) and covers a wide range of SSC concentrations from 0.1 to 5,760 mg/l (mean = 38.04 mg/l).​.

To maximize generalizability and minimize overfitting, Prum et al^23^. trained the XGBoost algorithm using global spatial-temporal cross validation, feature selection, and hyperparameter tuning, and used only optical features such as spectral reflectance and water color (Hue, Saturation, and Value). The relationship between SSC and​ Rs is affected by the co-varying signal from optical active constituents such as SSC, chromophoric dissolved organic matter and Chlorophyll-a. Including HSV color space features in the model helps disentangle SSC signal from the signal mixture and informs the model when predicting SSC in rivers not included in the training data. XGBoost was chosen by comparison with published algorithms and also XGBoost often outperforms other data-driven algorithms for Earth observations, such as remote sensing of water quality^52–54^. There were no performance differences in the SSC model between Landsat satellites suggesting successful harmonization of satellite Rs as well as spatial-temporal cross validation (Fig. 1). Extensive evaluation showed the model’s ability to capture spatial and temporal SSC variability in rivers^11^. Figure 2a presents the global distribution of long-term mean SSC, while Fig. 2b shows the corresponding spatial variability (standard deviation). While a global model is preferred for coherent SSC estimates over time and space, there will naturally be greater uncertainty in global models compared to local models. The major uncertainty in our model is overestimation in rivers with low SSC conditions (< 5 mg/L) Prum et al^23^..

As our focus here is on trends over time, we also validated rates of change over time comparing Sen’s Slope of annual mean satellite-derived SSC vs. annual mean field SSC over time. We identified all field monitoring stations in our model test set that had long-term SSC records suitable for quantifying rates of change using the Mann-Kendall test and Sen’s slope. After filtering only monitoring stations with a statistically significant trend (*p* < 0.05), we identified 62 monitoring stations largely in the US with one in Europe and two in East Asia. Rates of change quantified from satellite and field derived SSC showed strong agreement (*r* = 0.81, Figure S2), suggesting GloRivSed is capable of detecting trends and quantifying rates of changes that are comparable to long-term field measurements.

### Data analysis

We evaluated trends in mean annual SSC using the non-parametric Mann-Kendall test, chosen for its robustness against outliers^55,56^. For each river reach represented by SWORD centerlines, we calculated the mean annual SSC by averaging all available SSC data within each year. To provide a representative measure of annual SSC for trend robustness, we established a minimum threshold for data inclusion, analyzing 167,174 river reaches with at least 216 total SSC observations (equivalent to at least six observations per year), distributed across multiple seasons, and with time series spanning at least 15 years. These thresholds ensure that each time series contains sufficient temporal coverage to support statistically meaningful Mann–Kendall trend detection and reduce the influence of sparsely sampled reaches. We excluded satellite-derived SSC data from 1991 to 1994 to avoid potential artifacts introduced by the Mt. Pinatubo eruption, which affected atmospheric conditions and satellite signal quality during that period^13^. Notably, 75% of total river reaches had time series data extending 15 years or more. Significant trends were identified with a p-value threshold of < 0.05. The SSC trends were categorized as increasing trend, decreasing trend and no trend depending on if the slope was significantly positive, significantly negative, or not statistically significant.

 Our attribution analysis focused on 25 major river basins (> 600,000 sq.km), determining the percentage of river reaches with increasing, decreasing, and stable SSC trends in each river basin (Table S2). For each basin, we collected data on potential factors influencing SSC trends, including land cover change, dam regulation change, mean rainfall erosivity, dominant climate zone, lithology type, mean aridity, basin relief, and mean rainfall from the data sources linked in the data availability section. These candidate variables were selected based on well-established controls on sediment transport in river systems, including human regulation, land-surface change, climatic forcing, and basin physiographic characteristics^9,13,14^. Land cover change and dam regulation were treated as dynamic variables, capturing temporal variability, whereas all other factors were considered static, representing basin-scale long-term averages.

Land cover data was sourced from the European Space Agency (ESA) World Cover to calculate the percent changes in major land cover types in select 25 river basins between 2000 and 2022^57^. Because consistent global land cover products are only available for this period, these changes were used as an indicator of recent landscape change within the broader 1984–2022 SSC trend analysis. To facilitate basin-scale analysis, detailed land-cover classes were aggregated into nine broader categories representing dominant surface types: grassland, wetland, urban areas, water bodies, barren land, forest, shrubland, savanna, and cropland. For example, multiple forest subclasses (e.g., evergreen and deciduous needleleaf and broadleaf forests, and mixed forests) were grouped as forest, closed and open shrublands were combined as shrubland, and cropland mosaic classes were grouped with cropland. All analyses were conducted in Google Earth Engine (GEE), where annual gridded land cover maps were clipped to each basin using watershed boundaries. For each year, the area (in km²) of each land cover type within a basin was computed by summing the pixel area corresponding to that class. Percent change was then calculated by comparing class-specific areas in 2000 and 2022.

The temporal Degree of Regulation index (DOR) was used to quantify the extent of human influence on river discharge through dam regulation over time^58^. It was calculated as the change in reservoir storage capacity between 1984 and 2022, normalized by the long-term mean annual river discharge^32^. Dam storage capacity data were obtained from the Global Reservoir and Dam (GRanD) Database by the Global Water System Project (GWSP)^33^, summing the capacities of all relevant reservoirs within each basin. The corresponding mean annual river discharge values were derived from long-term hydrological datasets from Global Runoff Data Centre (GRDC)^59^. This temporal DOR index captures the dynamics of dam expansion over time.

$$\:Temporal\:DOR\:index=\frac{Change\:in\:total\:storage\:capacity\:(1984-2022)}{Mean\:annual\:river\:discharge\:at\:basin\:outlet}\:\times\:\:100\%$$

Mean rainfall erosivity for each basin was calculated using data from the Global Rainfall Erosivity Database (GloREDa)^15^. Dominant climate zones for each river basin were identified based on the Köppen-Geiger climate classification^60^. Lithology data was obtained from the Global Lithological Map^61^, and the percentage composition of major lithology types was calculated for each river basin. Basin-scale mean aridity indices were derived from the Global Aridity Index^17^, with values provided by the CGIAR-CSI Consortium for Spatial Information. Rainfall data were obtained from ERA5 Copernicus Climate^62^, and long-term average basin rainfall was calculated. Basin-average relief data were derived from the MERIT elevation dataset^63^. All datasets were processed using Google Earth Engine, where gridded layers were clipped to basin boundaries and aggregated using spatial averaging or class-based pixel summaries.

We performed Pearson correlation analysis between the percentage of increasing or decreasing SSC trends in river basins and potential influencing factors (Table S1) to identify candidate variables for regression analysis. We used multiple linear regression (MLR) to interpret the relative influence of drivers over the extent of SSC change. MLR was conducted separately for the fraction of decreasing and increasing SSC trends across basins. Independent variables were iteratively removed based on their statistical significance (p-value) and multicollinearity diagnostics. Multicollinearity among predictors was evaluated using the variance inflation factor (VIF), and variables with VIF values greater than 5 were removed to reduce redundancy among explanatory variables. The final model retained only variables with statistically significant contributions to the model’s explanatory power. Independent variables included temporal and static human, climatic, and geologic factors. Examples of human factors include change in land use and dams. Examples of temporal and static climatic factors include precipitation trends and mean rainfall erosivity respectively. Examples of geologic factors include lithology cover and topographic relief (Table S1). To aid interpretation, we identified the dominant climate type^60^ that accounts for the largest fraction of the basin area. To test if basin-scale relationships are consistent across spatial scales, we extended the correlation analysis to nested sub-basins (*n* = 214).

## Supplementary Information

Below is the link to the electronic supplementary material.

Supplementary Material 1

## Data Availability

The Global River Sediment Database (GloRivSed) database contains surface suspended sediment concentrations (SSC) derived from Landsat 5, 7, and 8 Level 1 Collection 1 surface reflectance from all rivers in the world that are ~ 60 meters wide or greater^9^ https://doi.org/10.5281/zenodo.15485524. Private Link to Zenodo Land cover data were from European Space Agency (ESA) WorldCover^52^ https://pure.iiasa.ac.at/id/eprint/18478/. Dam data were collected from Global Reservoir and Dam (GRanD) Database^28^ https://www.globaldamwatch.org/grand/. Hydrological datasets were from Global Runoff Data Centre (GRDC)^54^ https://www.researchgate.net/profile/Pete-Falloon/publication/252683891_New_Global_River_Routing_Scheme_in_the_Unified_Model/links/56b05e5e08ae8e37214d7b2a/New-Global-River-Routing-Scheme-in-the-Unified-Model.pdf. Rainfall erosivity data were from Global Rainfall Erosivity Database (GloREDa)^15^ https://esdac.jrc.ec.europa.eu/content/global-rainfall-erosivity. Climate classes were referred from Köppen-Geiger climate classification^55^ https://www.gloh2o.org/koppen/. Lithology data were from Global Lithological Map^56^ https://doi.pangaea.de/10.1594/PANGAEA.788537. Aridity data were from Global Aridity Index^57^ https://figshare.com/articles/dataset/Global_Aridity_Index_and_Potential_Evapotranspiration_ET0_Climate_Database_v2/7504448/5. Rainfall data were collected from Global Rainfall data ERA5 Copernicus Climate^58^ https://www.ecmwf.int/en/forecasts/dataset/ecmwf-reanalysis-v5. Elevation data were from MERIT elevation dataset^59^ http://hydro.iis.u-tokyo.ac.jp/~yamadai/MERIT_Hydro/.
