# Supplementary material for: Extent of sediment concentration trends associated with climate and human factors across global rivers
Source: Sci Rep. 2026 Apr 3;16:16062. doi: 10.1038/s41598-026-47267-2 (PMC13199382; doi:10.1038/s41598-026-47267-2)
Supplement: Supplementary file 1 — Supplementary Material 1 [file 41598_2026_47267_MOESM1_ESM.docx]

Supporting Information for

**Extent of sediment concentration trends associated with climate and human factors across global rivers**

Rajaram Prajapati^1,2^, John Gardner^1,3^, and Punwath Prum^1^

^1^Department of Geology and Environmental Science, University of Pittsburgh, Pittsburgh, PA, USA.

^2^SmartPhones4Water (S4W), Chico, CA, USA.

^3^Department of Earth, Marine and Environmental Sciences, University of North Carolina at Chapel Hill, NC, USA.

Corresponding author: Rajaram Prajapati ([livaraja08@gmail.com)](about:blank)

**Contents of this file**

Figures S1 to S6

Tables S1 to S2

**Figures**


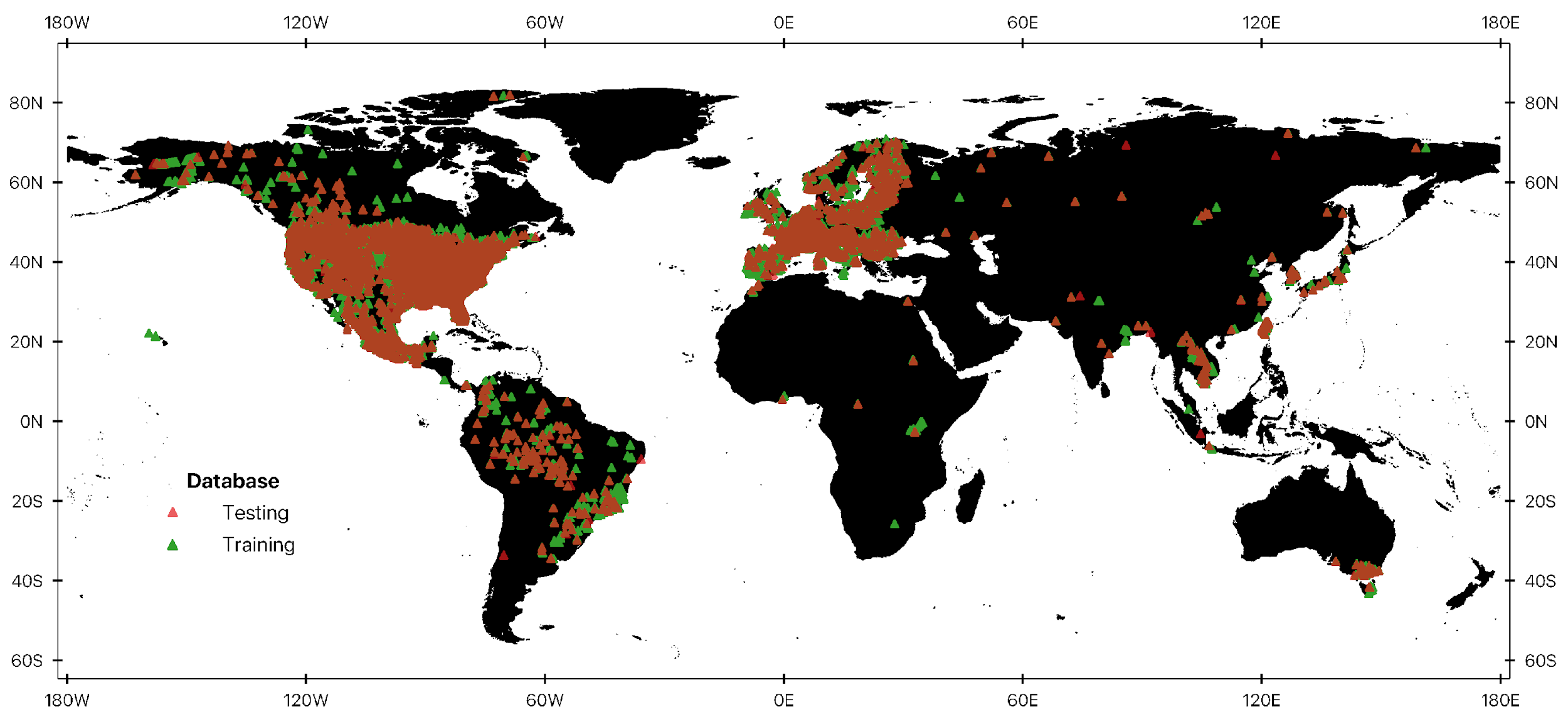


**Figure S1**. Global map of locations of training and testing data. The map was generated by the authors using R (version 4.5.3; <https://www.r-project.org/>) and does not require any permissions.


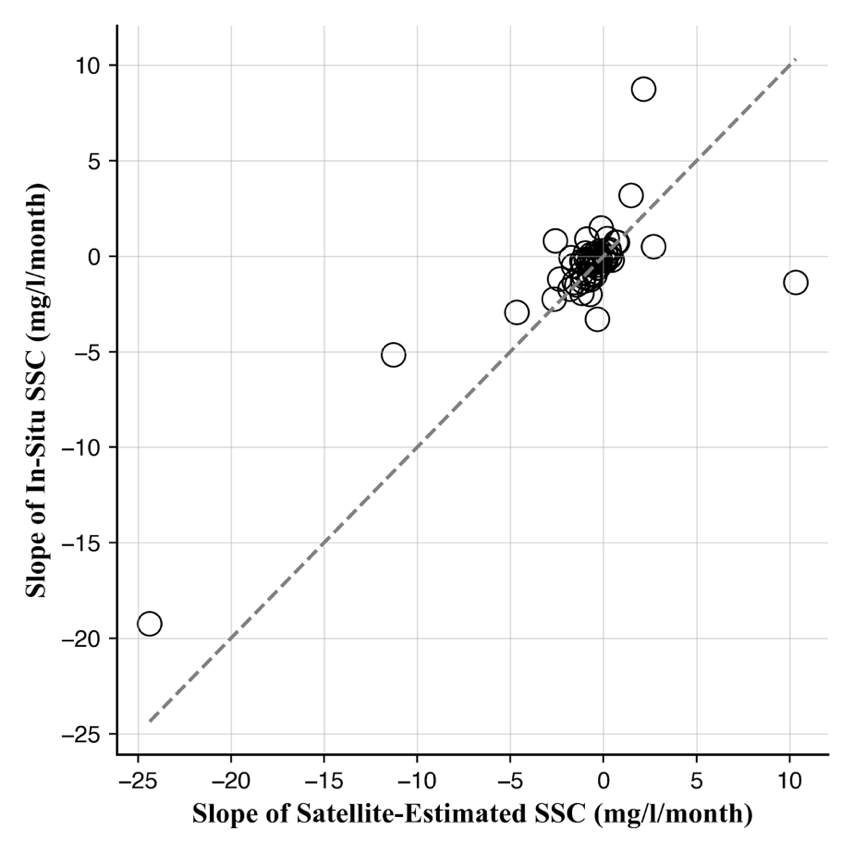


**Figure S2.** Comparison of Sen’s slope estimates for SSC trends derived from satellite-based predictions and in-situ observations across 62 long-term monitoring stations (each with >10 months of SSC data in the test set). The strong agreement (Pearson’s correlation = 0.81) indicates the reliability of satellite-derived trend detection. The dashed grey line denotes 1:1 line.

*
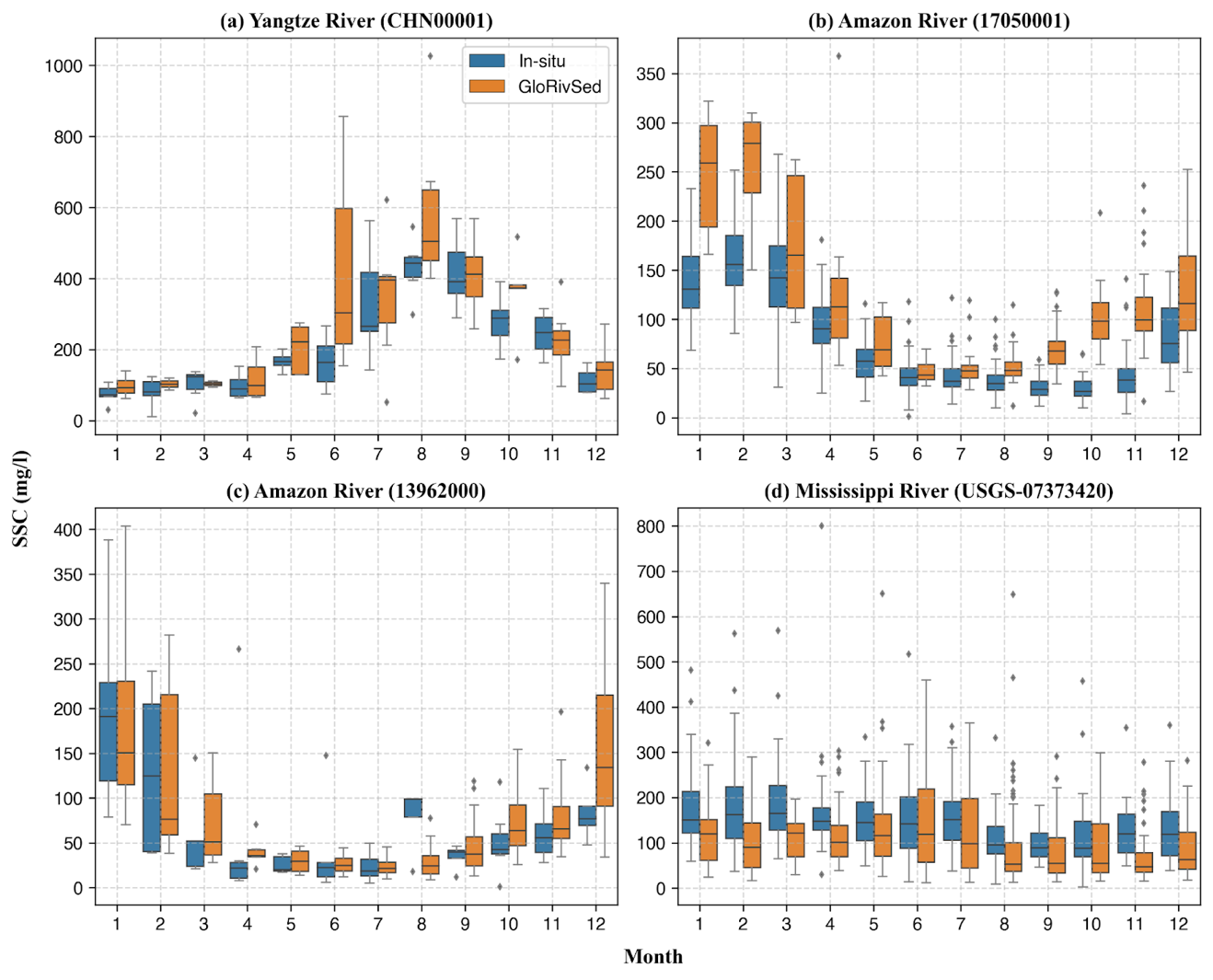
*

**Figure S3.** Monthly suspended sediment concentration (SSC) at four different sites comparing in-situ measurements (blue) with satellite-derived GloRivSed estimates (orange). Satellite-based estimates generally capture seasonal patterns of sediment transport but diverge in magnitude and variability during peak sediment months.

*
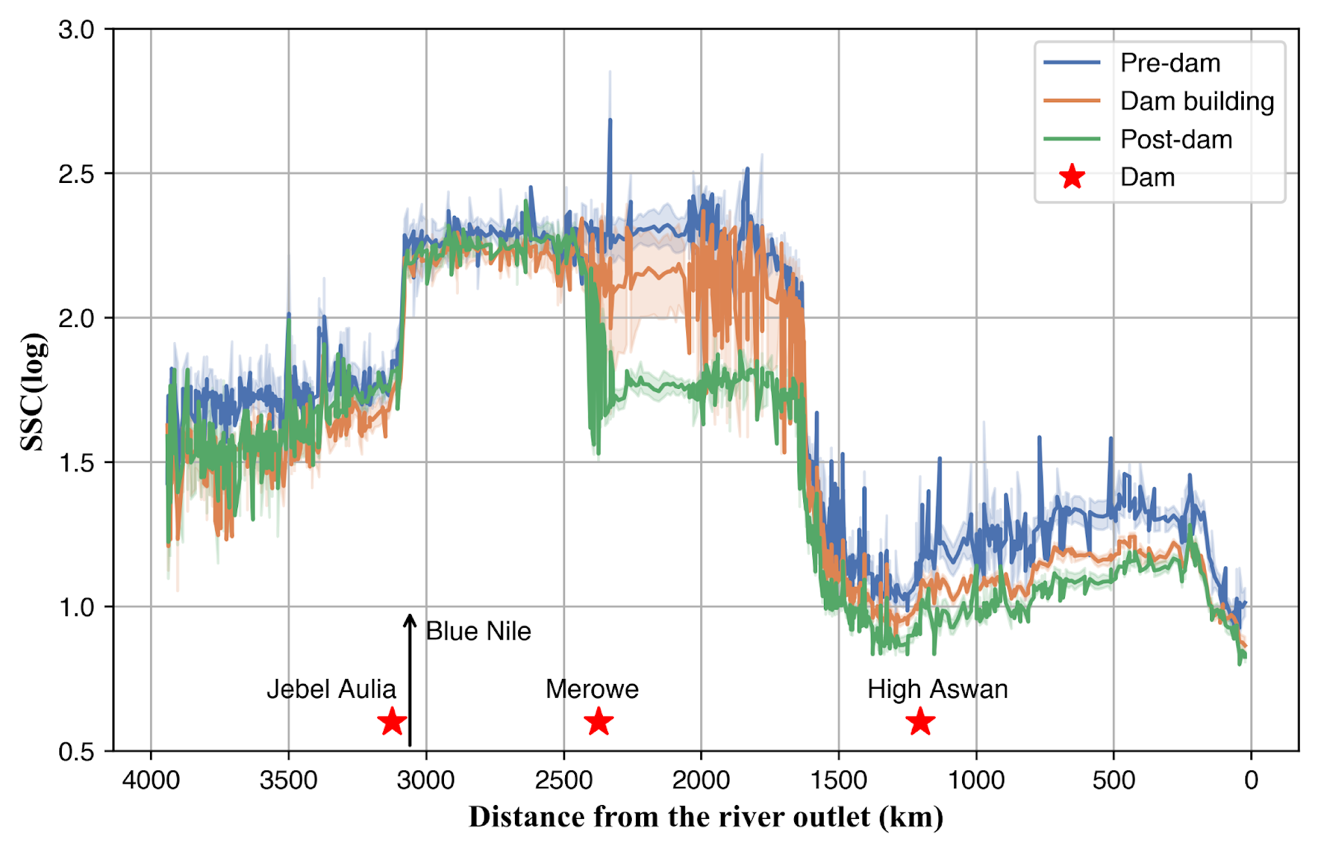
*

**Figure S4.** Longitudinal profile of log-transformed suspended sediment concentration (SSC) along the Nile River mainstem, highlighting temporal changes before, during, and after Merowe Dam construction (2004–2009). Lines represent reach-averaged SSC for each period. Red stars mark major dams, including Merowe, High Aswan, and Jebel Aulia. The upward black arrow indicates the confluence of the Blue Nile, where a notable increase in SSC is observed due to tributary sediment input.

**
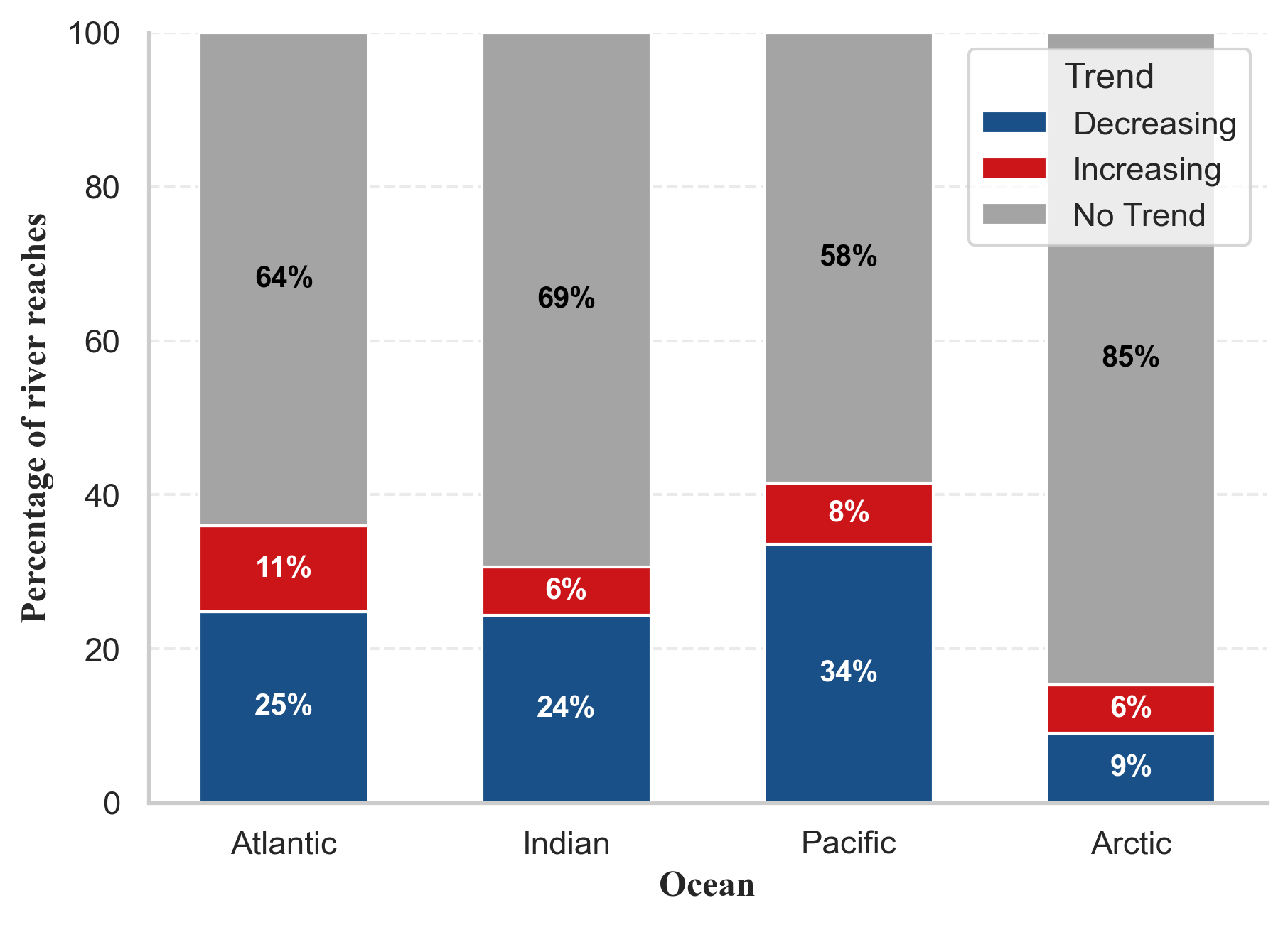
**

**Figure S5.** Distribution of SSC trends by ocean basin. River reaches are grouped by their receiving ocean (Atlantic, Indian, Pacific, and Arctic). Percentages indicate the fraction of reaches with decreasing, increasing, and no significant SSC trends. Atlantic and Indian basins show similar distributions, Pacific basins exhibit a higher proportion of decreasing trends, and Arctic basins are dominated by no significant trends.


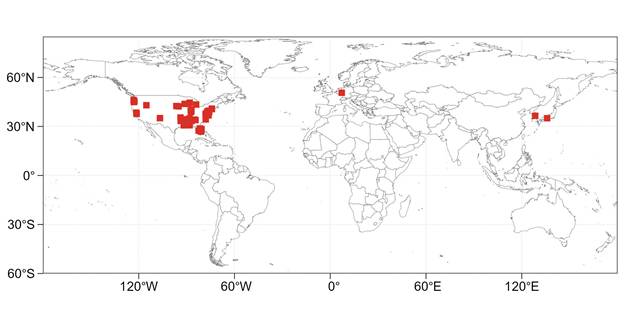


**Figure S6.** Locations of 62 sites with long-term SSC records over the study period. The map was generated by the authors using R (version 4.5.3; <https://www.r-project.org/>) and does not require any permissions.

**Tables**

**Table S1.** Correlation coefficients between potential influential factors and SSC related metrics (mean SSC, percentage of river reaches with decreasing SSC trends, and percentage of river reaches with increasing SSC trends). Positive and negative correlations indicate the direction of the association between each variable and SSC-related metrics. Statistically significant correlations are shown in bold.

| **Variables** | **Mean SSC**  **(mg/l)** | **Reaches with decreasing SSC trend (%)** | **Reaches with increasing SSC trend (%)** |
| --- | --- | --- | --- |
| Forest cover change (%) | 0.03 | **0.46** | -0.39 |
| Grassland change (%) | 0.21 | -0.22 | 0.1 |
| Shrubland change (%) | -0.1 | 0.22 | 0.2 |
| Cropland change (%) | 0.19 | 0.12 | -0.08 |
| Temporal DOR index (%) | 0.21 | **0.49** | -0.26 |
| Rainfall erosivity  ( MJmmha^-1^hr^-1^) | 0.13 | -0.07 | **0.62** |
| Aridity index | **-0.43** | -0.21 | **0.46** |
| Relief (m) | 0.36 | 0.05 | 0.14 |
| Mean annual rainfall (mm) | -0.07 | -0.04 | **0.52** |
| Rainfall trend (mm/year) | -0.23 | -0.30 | -0.28 |
| Unconsolidated sediment (%) | **0.59** | 0.25 | 0.02 |
| Carbonate sediment (%) | **-0.33** | 0.21 | -0.36 |

**Note:** *We found a statistically significant positive correlation (r = 0.61) between forest cover gain and the proportion of reaches with decreasing SSC trends in basin level, and a negative correlation (r = –0.51) between forest cover loss and increasing SSC trends at the basin level (when excluding 8 basins with minimal, <5% forest cover).*

**Table S2.** Summary of SSC trends and climatic characteristics across select river basins.

| **S.N.** | **Basin** | **Area**  **(sq. km.)** | **Mean SSC**  **(mg/l)** | **Dominant Climate** | **SSC**  **Percent**  **decrease** | **SSC**  **Percent increase** | **Mean**  **Annual Rainfall** | **Continent** |
| --- | --- | --- | --- | --- | --- | --- | --- | --- |
| 1 | Amazon | 5912923 | 97.70 | Tropical | 15.93 | 11.76 | 2374.44 | S. America |
| 2 | Amur | 2238973 | 46.24 | Cold | 23.98 | 9.95 | 613.07 | Asia |
| 3 | Congo | 3705222 | 72.45 | Tropical | 20.74 | 15.01 | 1565.87 | Africa |
| 4 | Danube | 795318 | 43.99 | Temperate | 39.74 | 3.41 | 872.87 | Europe |
| 5 | Euphrates | 935570 | 60.15 | Arid | 69.05 | 0.80 | 385.60 | Europe |
| 6 | Ganges | 1584745 | 53.58 | Temperate | 31.71 | 4.83 | 1583.07 | Asia |
| 7 | Hwang Ho | 962987 | 180.33 | Arid | 45.48 | 3.74 | 548.18 | Asia |
| 8 | Indus | 864624 | 83.97 | Arid | 37.86 | 2.25 | 680.99 | Asia |
| 9 | Kolyma | 1614054 | 33.84 | Cold | 1.43 | 9.25 | 369.69 | Asia |
| 10 | Lena | 2453648 | 24.68 | Cold | 9.87 | 6.33 | 484.58 | Asia |
| 11 | Mackenzie | 1795627 | 79.70 | Cold | 12.21 | 4.54 | 492.24 | N. America |
| 12 | Mississippi | 3240617 | 77.87 | Temperate | 39.51 | 1.94 | 837.97 | N. America |
| 13 | Murray | 1055416 | 133.91 | Arid | 33.48 | 1.79 | 461.79 | Australia |
| 14 | Nelson | 1106517 | 40.53 | Cold | 13.41 | 16.81 | 590.42 | N. America |
| 15 | Niger | 2122996 | 128.86 | Tropical | 6.49 | 18.07 | 521.77 | Africa |
| 16 | Nile | 3057772 | 106.30 | Arid | 40.07 | 7.46 | 741.69 | Africa |
| 17 | Ob | 3088705 | 34.46 | Cold | 14.60 | 4.38 | 562.57 | Asia |
| 18 | Orange | 977324 | 97.62 | Arid | 67.55 | 0.27 | 391.69 | Africa |
| 19 | Parana | 2646303 | 99.76 | Temperate | 45.08 | 8.28 | 1328.40 | S. America |
| 20 | St Lawrence | 1053296 | 20.10 | Cold | 31.84 | 4.05 | 985.56 | N. America |
| 21 | Volga | 1404137 | 26.76 | Cold | 63.90 | 0.29 | 694.87 | Europe |
| 22 | Yangtze | 1924625 | 51.12 | Temperate | 56.03 | 3.70 | 1354.99 | Asia |
| 23 | Yenisey | 2505668 | 19.38 | Cold | 14.87 | 4.17 | 586.63 | Asia |
| 24 | Yukon | 832819 | 64.63 | Cold | 7.563 | 8.00 | 514.20 | N. America |
| 25 | Zambezi | 1378103 | 64.29 | Temperate | 27.18 | 6.47 | 1051.44 | Africa |
